# Twelve Years of Scientific Production on Medline by Latin American Spine Surgeons

**DOI:** 10.1371/journal.pone.0087945

**Published:** 2014-02-05

**Authors:** Asdrubal Falavigna, Ricardo Vieira Botelho, Alisson Roberto Teles, Pedro Guarise da Silva, Delio Martins, Juan Pablo Guyot, Alvaro Silva Gonzalez, José Maria Jiménez Avila, Helton Luiz Aparecido Defino

**Affiliations:** 1 Department of Neurosurgery, University of Caxias do Sul, Caxias do Sul, RS, Brazil; 2 Department of Neurosurgery, Hospital do Servidor Público Estadual Francisco Morato de Oliveira, São Paulo, SP, Brazil; 3 Department of Neurosurgery, Hospital São José of the Santa Casa de Porto Alegre, Porto Alegre, RS, Brazil; 4 Department of Orthopedics, Federal University of São Paulo, Sao Paulo, SP, Brazil; 5 Department of Orthopedics, Hospital Universitario Fundación Favaloro, Buenos Aires, Argentina; 6 Department of Orthopedics, Universidad del Desarrollo, Santiago de Chile, Chile; 7 Department of Orthopedics, Centro Médico Nacional de Occidente, Guadalajara, Mexico; 8 Department of Orthopedics, University of São Paulo, São Paulo, SP, Brazil; Katholieke Universiteit Leuven, Belgium

## Abstract

**Background:**

Despite the small contribution of LA in the Science Citation Index (SCI), a growing contribution by LA research to international literature has been observed in recent years.

**Study Design:**

Systematic review.

**Purpose:**

To evaluate the scientific contribution of Latin American (LA) Spine Surgeons in the last decade.

**Methods:**

A literature search of publications by LA spinal surgeons on topics concerning the spine or spinal cord was performed using an online database; Pubmed.gov. The results were limited to articles published from January 2000 to December 2011. The quality of the publication was evaluated with the journal impact factor (IF), Oxford classification and number of citations.

**Results:**

This study comprised 320 articles published in the Medline database by LA spine surgeons from 2000 to 2011. In recent years, there has been an increase in the number of publications by LA spine surgeons. It was observed that 38.4% of LA papers were published in LA journals. 46.6% of the articles were published in journals with an IF lower than 1, and there was no statistically significant difference in the number of articles published in journals with a higher IF during the period. Linear-by-linear association analysis demonstrated an improvement in the level of evidence provided by LA articles published in recent years.

**Conclusions:**

This study showed a growth in the number of publications in last 12 years by LA spinal surgeons. It is necessary to discuss a way to increase quantity and quality of scientific publications, mainly through a better education in research.

## Introduction

The researcher must be committed to publishing the results of research, and also choose the appropriate means to make the scientific production visible in order to share knowledge with the community and to ensure the best medical decision and therapy for the patient [1]–[3]. The articles published about spine pathologies have been very useful, providing major information to improve diagnosis and choice of treatment. This information is even more important when it comes from the different continents, because it shows that information can be provided and applied by physicians worldwide to solve medical problems encountered daily.

The visibility of the papers is achieved by their publication in high-quality journals that are readily available to be consulted [4]–[5]. The Institute of Scientific Information (ISI) and the Science Citation Index (SCI), are the instruments most used by researchers and specialists to access bibliographic information and citations [6]. At the end of the 90s, 14 Latin American (LA) and Caribbean journals were registered at SCI and LA generated only 1.4% of the 70,000 scientific papers produced worldwide [7].

Despite the small contribution of LA to SCI, an increase of 1% to 1.8% in scientific publications was observed in LA and in several countries between 1986 and 1991 [9]–[11]. These increases were below expectations and the main reasons for this were culture, lack of economic resources and low qualification of the researchers [8].

The purpose of this article is to evaluate the scientific contribution of the LA spine surgeons in 12 years and to discuss the strategies that can be used to increase number and quality of publications on this subject.

## Materials and Methods

A literature search of publications by the LA spine surgeons on spine or spinal cord topics was performed using an online database: Pubmed.gov (http://www.ncbi.nlm.nih.gov/pubmed/). The results were stored and analyzed at the Laboratory of Clinical Studies and Basic Models of Spinal Disorders of the University of Caxias do Sul. The results were limited to articles published from January 2000 to December 2011. The articles could be published in all spinal journals regardless of country where the journals were published, but the journal must be indexed in the Medline database. The search terms used were Argentina OR Bolivia OR Brazil OR Chile OR Colombia OR Costa Rica OR Cuba OR Dominican Republic OR Ecuador OR El Salvador OR Guatemala OR Guiana OR Haiti OR Honduras OR Mexico OR Nicaragua OR Panama OR Paraguay OR Peru OR Puerto Rico OR Surinam OR Uruguay OR Venezuela* AND (“spine” OR “spinal diseases” OR “spinal cord” OR “spinal cord diseases” OR “vertebroplasty” OR “arthrodesis” OR “diskectomy” OR “foraminotomy” OR “laminectomy” OR “denervation” OR “back injuries”).

**Figure 1:**
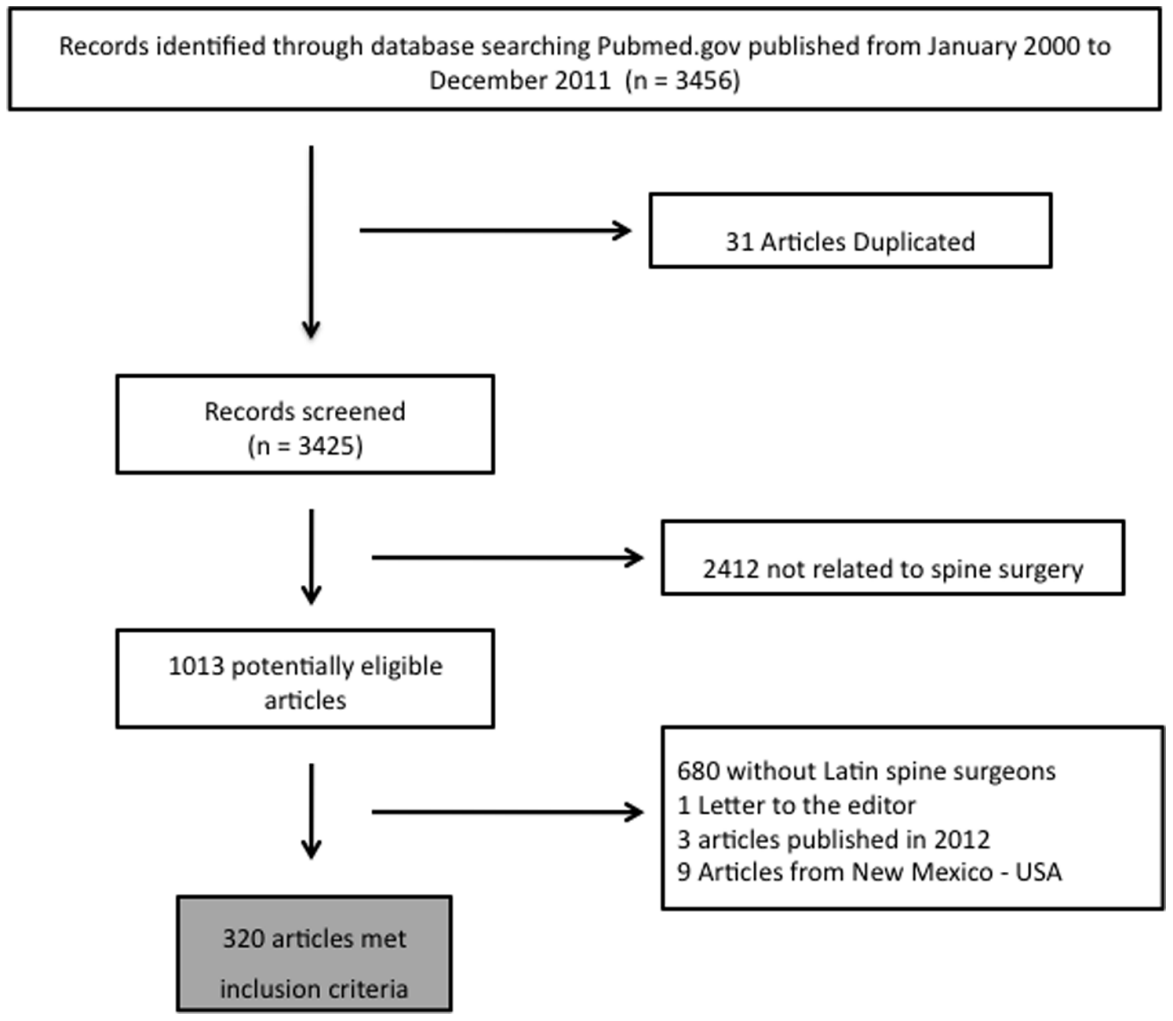
Literature review of publications by the Latin American spine surgeons on spine or spinal cord topics using an online database of Pubmed.gov, during the period from January 2000 to December 2011 with the total numbers and the exclusion criteria.

Based on title and abstract we excluded articles that were not published in the defined period (2000–2011), subject not related to spine pathology. The selected papers were further analyzed and excluded the letters to the editor, and papers in which no LA spine surgeons were found as authors (Figure 1).

The LA spine surgeons were defined as spine surgeons from orthopedic or neurosurgical specialties who lives and work in LA Countries. The presence of LA spine surgeons as authors was first defined according to their affiliation. When this information was not presented in the paper, the curriculum vitae was looked at on the internet and/or their affiliation was requested via an e-mail sent to the correspondent author.

An excel sheet was done with the main information of the selected papers, such as the authors full names, year of publication, journal, country of journal, impact factor (IF) of the journal, number of citations, type of publication, level of evidence (LOE) provided by the article, presence or not of a foreign author, and main pathology studied. This avoids the risk of duplicate papers.

Those variables were analyzed by two independent authors and in case of discordance, the two reviewers discussed the matter together and found a consensus. Few discordances were found after the analyses, suggesting high inter reviewer reliability of the evaluations.

The quality of the journals was evaluated with the IF of the journal. This was assessed using the classification published in 2010 in the Journal of Citation Reports (JCR) [12]. The quality or LOE of the articles was evaluated using the Oxford classification, [13] as well their number of citations per article. The type of article was divided into clinical studies, case reports, experimental studies, case series, reviews, technical reports and meta-analyses.

Statistical analyses were conducted with SPSS 20.0. Categorical variables were presented as number and proportion. Linear-by-linear association tests and ANOVA test were conducted in order to verify improvement in quality of publications in the 12 years period.

## Results

A total of 3,456 articles were identified after the Medline search. Most of the articles were excluded based on information provided in the title and abstract. Reasons for exclusion of the remaining articles are shown in Figure 1. This study comprised 320 articles published in the Medline database by LA spine surgeons from 2000 to 2011.

**Figure 2:**
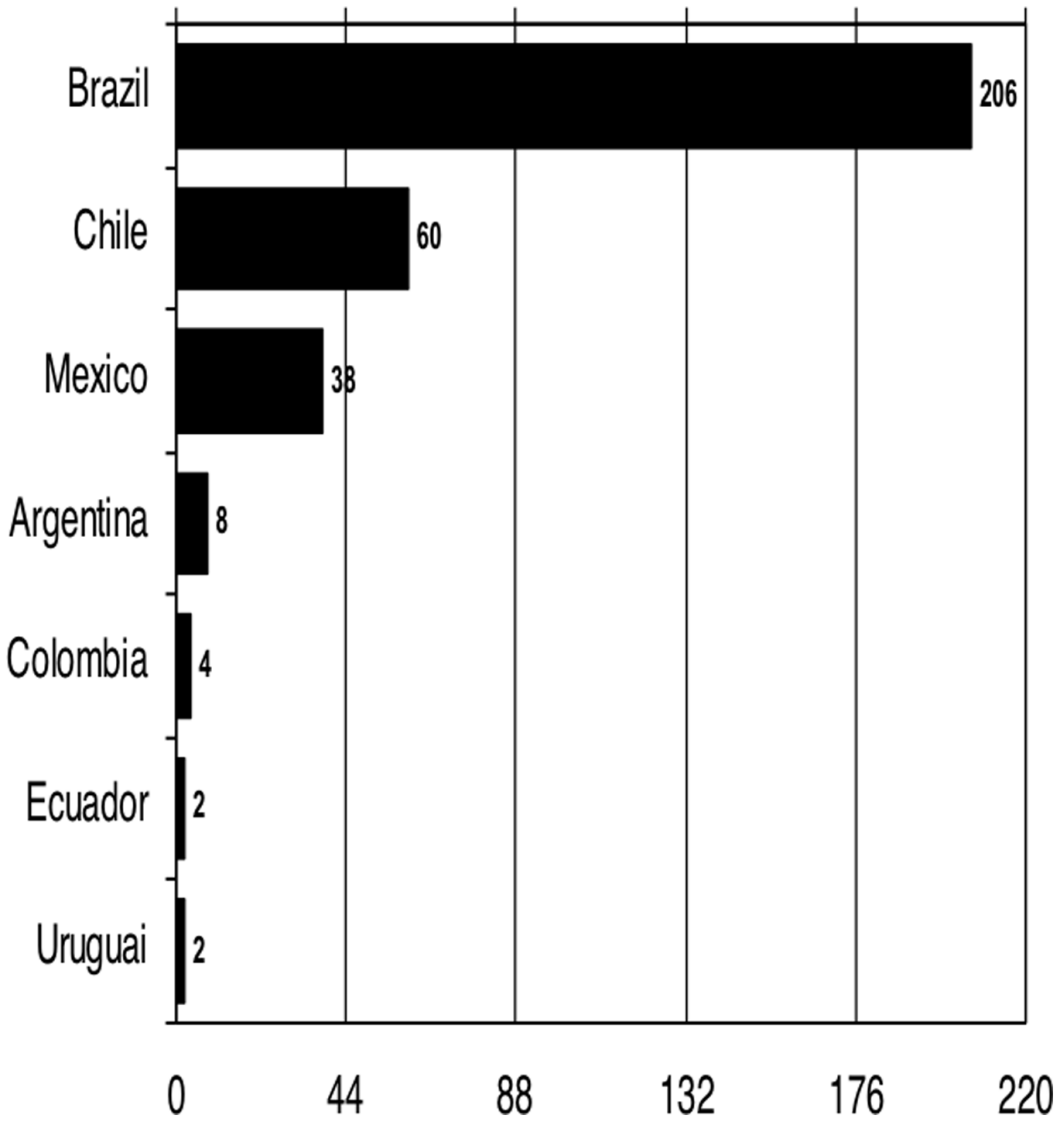
Number of papers according to country.

Only 7 of analyzed LA countries have published papers in Medline during the study period. Most of the papers were produced by Brazilian spine surgeons (64.4%), followed by Chile (18.8%) and Mexico (11.9%) (Figure 2). A participating foreign (non-Latin American) coauthor was identified in only 8 of 320 papers (2.5%).

**Figure 3:**
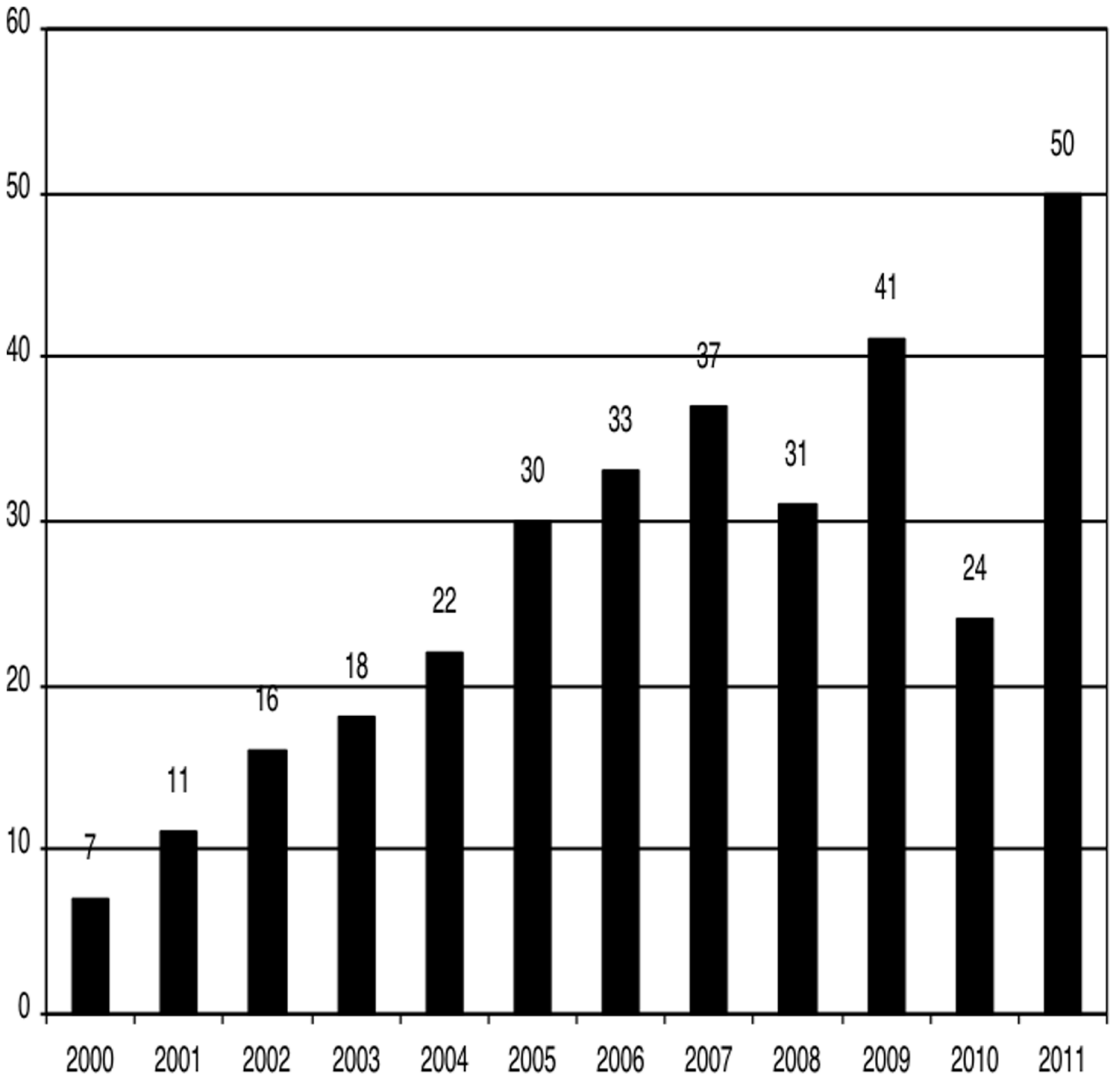
Number of papers according to year of publication.

Figure 3 shows the number of papers according to the year of publication. An increasing number of publications has been observed in the last few years: 45.3% of the articles were published between 2008 and 2011, 38.4% between 2004 and 2007, and only 16.3% between 2000 and 2003. Compared to the period of 2000 and 2003, a growth of 180% in the number of publications by LA spine surgeons was observed in the last 4 years, despite the unexplained reduction in 2008 and 2010.

**Figure 4:**
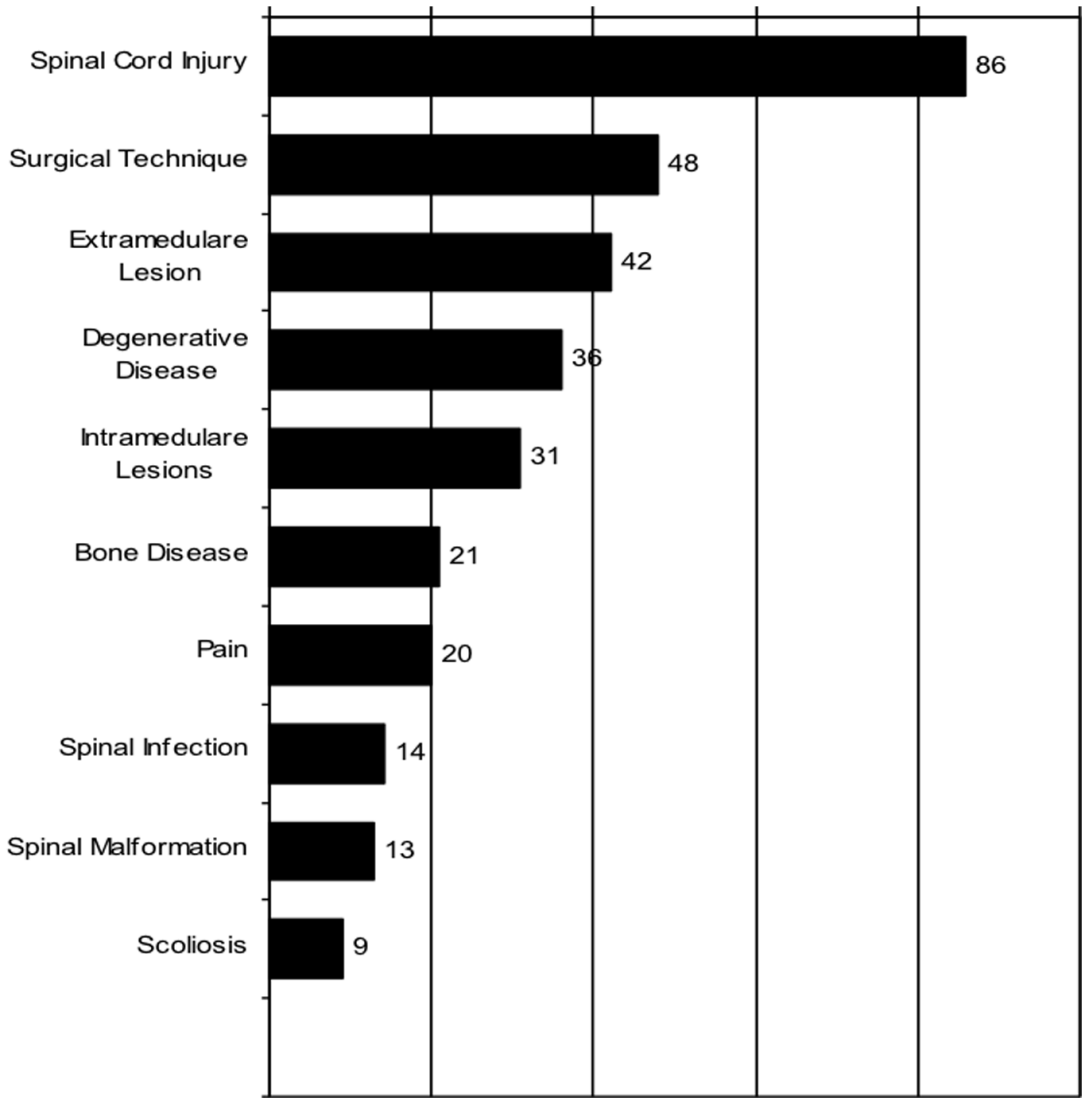
Number of papers according to subject.

Clinical studies (N = 121; 37.8%) and case reports (N = 76; 23.8%) were the most common types of articles. The remaining papers included experimental studies (N = 47; 14.7%), case series (N = 38; 11.9%), narrative reviews (N = 23; 7.2%), anatomical studies (N = 11; 3.4%), and systematic reviews or metanalysis (N = 4; 1.3%). Figure 4 shows the main subject of the articles.

**Figure 5:**
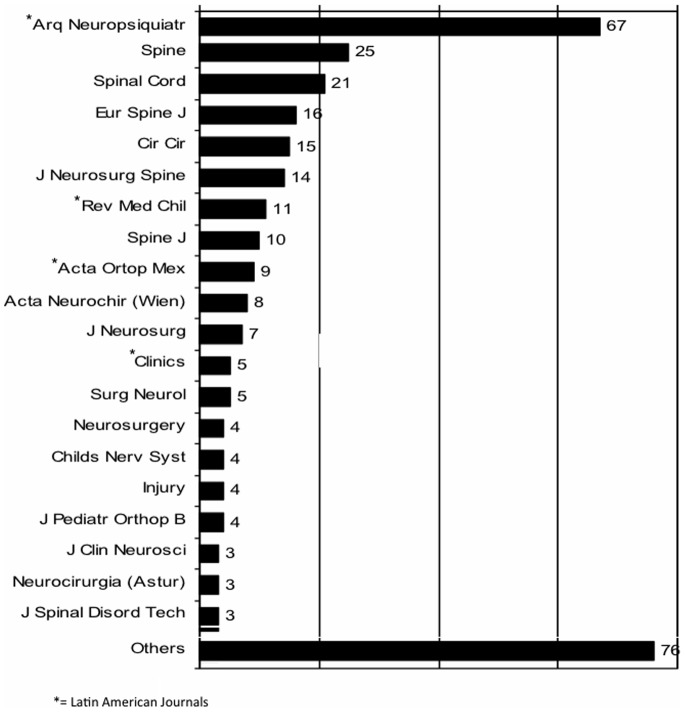
Number of papers according to journal.

Figure 5 shows the journals that most often published LA spine papers in the period. Journals that published less than 3 LA articles were included in the category “others”. It was observed that 38.4% (N = 123) of LA papers were published in journals from a LA country. The remaining articles were published in North American or European Journals (N = 197; 61.6%).

**Figure 6:**
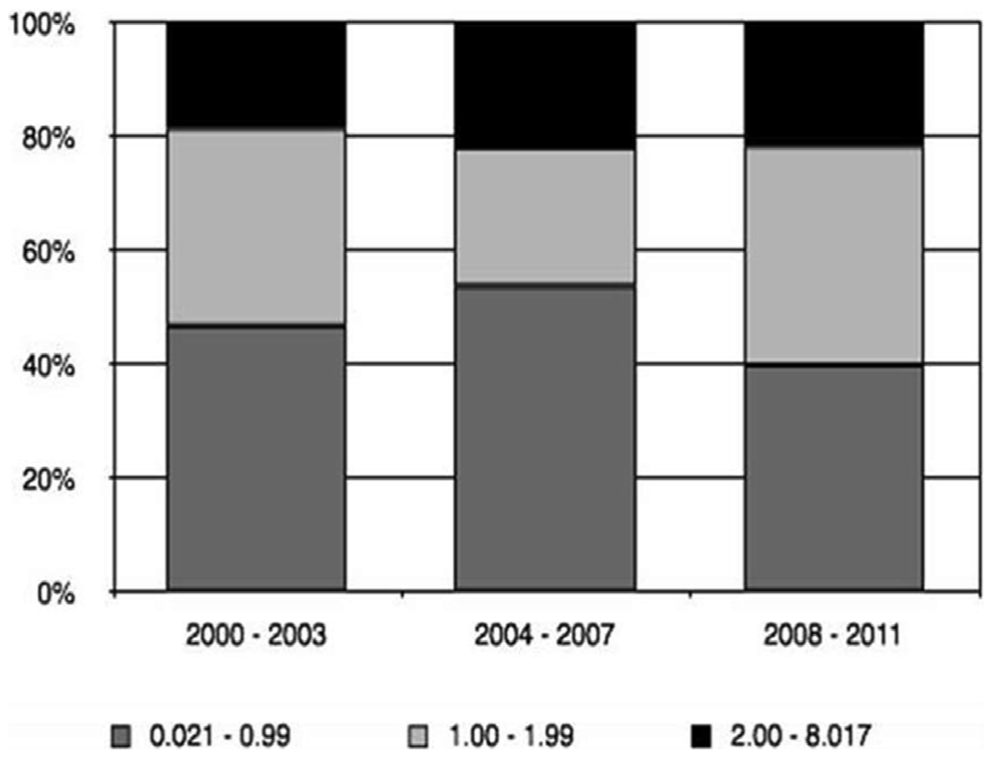
Proportion of papers according to impact factor of the journal during the period of evaluation.

The IF of the journals in which LA papers were published varied from 0.021 to 8.017. 46.6% of the articles were published in journals with an IF lower than 1, 32.3% with an IF between 1 and 2, and 21.1% higher than 2. There was no statistically significant difference in the number of articles published in journals with a higher IF during the period (Figure 6; Chi-square: 6.609; P = 0.158).

Analysis of the Oxford classification demonstrated that most LA papers provided LOE 4 (N = 171, 53.4%), followed by LOE 5 (N = 56, 17.5%), 2 (N = 61, 19.1%), 3 (N = 29, 9.1%), 1 (N = 3, 0.9%). Linear-by-linear association analysis demonstrated an improvement in the LOE provided by LA articles published in recent years (Linear-by-linear association: 12.955; P<0.0001). Table 1 presents the evolution of Oxford classification between 2000 and 2011.

**Table 1:** The quality of the articles evaluated by Oxford classification.

OxfordClassification	2000–2003	2004–2007	2008–2011	Total
1	0	1	2	**3**
2	6	18	37	**61**
3	3	8	18	**29**
4	32	74	65	**171**
5	11	21	24	**56**
**Total**	**52**	**122**	**146**	**320**

The analysis of number of citations per article could be performed in 296 papers, the remaining 24 were not found in the database of the Journal of Citation Reports. The 296 articles received a total of 1,557 citations. The number of citations per article varied from 0 (26.7%) to 39 (0.3%). The median of citations per article included in the analysis was 3 (P25: 0–P75: 8, mean: 5.26, standard deviation: 6.61). The mean number of citations per year of publication did not change during the study period. The mean number of citations per year was 0.94 (±0.75), 1.11 (±1.26), and 0.91 (±1.37) for articles published from 2000 to 2003, 2004 to 2007, and 2008 to 2011, respectively (ANOVA test: P = 0.442).

## Discussion

An increasing number of publications has been observed in the last few years with progressive increasing in the proportions of papers with a higher LOE, although there was no statistically significant difference in the number of articles published in journals with a higher IF during the period.

A question commonly asked by LA researchers in the 70′s, when LA publications began to be accessible to the international scientific community due to their inclusion in international databases, was whether it would be possible to attain the levels of scientific production of more advanced countries [8]. At the time, analyses demonstrated that the presence of LA publications in SCI was very low, with only 1% of the total publications in 1978 [14]. However, LA research contributions increased from 9,622 papers in 1990 to 22,589 papers in 2000, being responsible for 3% to 4% of the publications worldwide [8], [14]. Another analysis also demonstrated a growing contribution by LA research to international literature in recent years [15]–[16].

Specifically about spine and spinal cord diseases, the LA spine surgeons published a total of 320 papers indexed in Medline between 2000 and 2011. Our analyses demonstrated a growth of 180% in the number of LA papers during the last 4 years, compared with the first 4 years of evaluation. The increase in LA publication in surgical fields can be explained by the increase in opportunities for international exchange, the widespread use of the internet and more surgeons dedicated to basic and clinical studies [17].

Despite the growth in the number of publications by LA spine surgeons, the contribution of these countries to the global literature is still very low [16]. There are many factors involved in the low production by LA spine surgeons, such as the predominance of an oral culture, unfamiliarity with the English language and other types of biases (bias towards nationality) that can influence the approval rate [18]–[19], belief in lack of interest on the part of researchers from central countries in relation to research done in the periphery, the ephemeral life of local magazines, absence of economic resources, lack of trained personnel, and the low quality of the publications [8]. It is possible that LA publications are in a vicious cycle, where most of the national publications have no international prestige and the national journals do not have impact papers because regional scientists publish their best results abroad [20]. Our analyses identified only 8 papers with international collaboration (2.5%).

Brazil is the largest LA country and contributed 64.4% of the 320 identified spine papers during the last decade. These results are related to some efforts by federal and state agencies to promote Brazilian scientific production. These efforts contributed to the change in the worldwide ranking of indexations from the 22^nd^ position in 1998 to 13^th^ in 2008 [21]. There has been a growth in the input of development resources for research in the country, as well as a continuous demand for better individual performance from the researchers; a growth in the number and amount of federal scholarships has been observed in recent years; also, the creation of the Qualis Program of Coordenação de Aperfeiçoamento de Pessoal de Nível Superior (CAPES), which classifies foreign and Brazilian journals to guide the CAPES evaluation. At the same time, a better qualification of researchers has been identified by the increasing number of people with graduate degrees, especially doctorates, responsible for most of the Brazilian scientific production.

In Mexico the Consejo Nacional de Ciencia y Tecnología (CONACyT) is responsible for supporting policies and programs to incentivize the development of scientific research. The idea is to encourage the participation of organizations to promote activities of basic and applied scientific research in order to strengthen the national system of science, technology and innovation. In this sense, programs that CONACyT has implemented to boost scientific development include the development of a national system of researchers, topics of research networks of interest to the country, the opportunity for post-doctoral fellowships and financial support for research projects [22]. Despite government efforts, a contribution of 0.7 articles relating to spinal disorders subjects was found in approximately 3,500 papers/week involving trauma and general orthopedics. Similarly to general orthopedic publications, publications on the subject of spinal surgery have also decreased [23]. The main issue in Mexico is that the dissemination of science takes place primarily in national journals that have low international representation, and the few relevant articles are published abroad not improving the quality of national journals [20].

Although our data clearly demonstrate an increasing number of LA publications on spine disorders in 12 years, the IF of journals that are publishing those papers and also the number of citations per year of publication did not change. Interestingly, we observed a growth in the number of papers providing higher LOE during the last years. Differently, Rothoerl et al [15] published an investigation about LOE in the neurosurgical literature in 1999 and reported that 4% of the literature provided LOE 1. Ten years later, Yarascavitch et al [16] reviewed and compared the LOE of clinical studies published in the same journals by Rothoerl et al [15] from October 2009 through September 2010. There has been a decrease in the proportion of higher LOE (1 and 2) [16]. Several publications within the neurosurgical literature have addressed the challenges of producing research with a higher LOE and have provided guidance to assist with this goal [24]–[26].

The main paradigms needed to perform research are financial support and principally knowledge, capacity, and training for research. In this sense, the goal established by the AOSpine international research group during the meeting in Dallas is the better qualification of spine surgeons and offering conditions for research training and exchanging knowledge. Specifically in LA, the goals of this entity are: to identify and stimulate possible research groups that have the capacity to participate in regional or multicenter projects; to promote joint scientific activities to allow the integration and exchange of experiences; to identify and use the strengths of each research center to provide exchange of training and capacity building of human resources; to enable multicenter and interregional research projects; and to hold competitive studies that will enable obtaining funds from development agencies and governmental and non-governmental entities. It is hoped that the above mentioned measures to be adopted will improve the quality and number of publications in Latin America.

This study showed a clear growth in the number of publications by LA spinal surgeons in 12 years. We believe that the main strategy used to increase the quantity and quality of scientific publications is to build a greater awareness of the need for improvement and better education for research. It is pressingly necessary for LA spine surgeons to show the excellence of their work, regardless of all the difficulties we face in our region.

## Supporting Information

Checklist S1 (DOCX)
