# Supplementary material for: Twelve Years of Scientific Production on Medline by Latin American Spine Surgeons
Source: PLoS One. 2014 Feb 5;9(2):e87945. doi: 10.1371/journal.pone.0087945 (PMC3914870; doi:10.1371/journal.pone.0087945)
Supplement: Checklist S1 — (DOCX) [file pone.0087945.s001.docx]

**PRISMA**


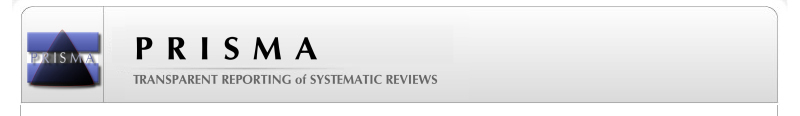


Full-text articles assessed for eligibility
(n = 1013)

Records excluded
(n = 2412)

Records screened
(n = 3425)

Records after duplicates removed
(n = 31)

Studies included to the analysis
(n = 320)

Full-text articles excluded, with reasons (n = 693)

- n=680 without Latin spine surgeons

- n=1 Letter to the editor

- n=3 articles published in 2012

- n=9 Articles from New Mexico - USA

Records identified through database searching Pubmed.gov published from January 2000 to December 2011
(n = 3456)

## Included

## Identification

## Eligibility

## Screening
